# Cyber Attacker Profiling for Risk Analysis Based on Machine Learning

**DOI:** 10.3390/s23042028

**Published:** 2023-02-10

**Authors:** Igor Kotenko, Elena Fedorchenko, Evgenia Novikova, Ashish Jha

**Affiliations:** Computer Security Problems Laboratory, St. Petersburg Federal Research Center of the Russian Academy of Sciences, 199178 Saint-Petersburg, Russia

**Keywords:** attacker profile, attacker model, attacker attribution, attributes, raw data, risk analysis, data analysis, machine learning, LSTM, bash commands

## Abstract

The notion of the attacker profile is often used in risk analysis tasks such as cyber attack forecasting, security incident investigations and security decision support. The attacker profile is a set of attributes characterising an attacker and their behaviour. This paper analyzes the research in the area of attacker modelling and presents the analysis results as a classification of attacker models, attributes and risk analysis techniques that are used to construct the attacker models. The authors introduce a formal two-level attacker model that consists of high-level attributes calculated using low-level attributes that are in turn calculated on the basis of the raw security data. To specify the low-level attributes, the authors performed a series of experiments with datasets of attacks. Firstly, the requirements of the datasets for the experiments were specified in order to select the appropriate datasets, and, afterwards, the applicability of the attributes formed on the basis of such nominal parameters as bash commands and event logs to calculate high-level attributes was evaluated. The results allow us to conclude that attack team profiles can be differentiated using nominal parameters such as bash history logs. At the same time, accurate attacker profiling requires the extension of the low-level attributes list.

## 1. Introduction

One of the essential components of cyber security risk analysis is an attacker model definition [1,2]. The specified attacker model, or attacker profile, affects the results of risk analysis, and further the selection of the security measures for the information system. Moreover, it can be used in cyber forensics tasks. Researchers have proposed various attacker models [3,4,5,6,7,8,9]. This study aims at the classification of the attacker models into low-level and high-level models depending on the type of attributes they consider. A high-level model operates with high-level attributes such as such as the goal, the location of the attacker (internal or external) or the complexity of the exploited vulnerabilities (low, medium or high) [3] to determine the the attacker type. A low-level model uses low-level attributes (or features) such as destination port, alert signature, source port, host, etc. [8,9] for the attacker definition. The high-level models allow us to determine the possible classes of attackers such as hackers, spies, terrorists, corporate raiders, professional criminals, vandals, and voyeurs [10,11,12], while the low-level models allow us to determine both an attacker class, a behaviour model and a specific malicious person or group of persons. It is especially essential for critical infrastructures, where the attacker has to be found by law enforcement because of the impact on citizens’ and society’s safety.

The first type of attacker model is usually used in the approaches to risk analysis based on attack graph analysis, while the second type of model is usually used in the approaches based on the hidden Markov model [13], fuzzy inference [14,15] and data mining methods including neural networks, statistics, etc. [16].

The second group of techniques looks more preferable as soon as it gives more accurate results based on the concrete measurements of the characteristics of the analyzed system under attack. It also allows us to determine the high-level attacker characteristics based on the low-level characteristics. Thus, the authors argue that an approach to attacker profiling that uses relations between features formed on the basis of the raw security-related data for representing attacker behaviour and attack development forecasting is promising for timely and efficient risk analysis and cyber attack counteraction. However, revealing the relationships between the high-level and low-level attributes is a rather challenging task that significantly limits the development of such an approach. There is also a lack of consistently labeled datasets that can be used to train a model to reveal such relationships.

In [17], the authors analyzed existing models of both classes and their application for risk analysis tasks. This paper extends the obtained results and presents them in the form of a taxonomy of the attacker model attributes that considers risk analysis techniques that use attacker models.

In [17], the authors formulated a set of questions related to the attacker model as follows:

1. how do we specify the attacker model?
2. how do we automatically calculate the values of attributes constituting the attacker model to determine the attacker profile using a non-expert technique based on the dynamic data gathered from logs and traffic during target system operation?
3. where do we get the appropriate initial data for the experiments?
4. do we really need an attacker model to analyze information security risks?

The preliminary results of the effort to answer the first question are presented in [18]. The authors also specified the requirements of the datasets for the experiments to answer the third question. This paper is the extended version of [18], which was presented at the International Conference on Risks and Security of Internet and Systems (CRiSIS) 2020 “Risks and Security of Internet and Systems”. It differs from the previous paper due to an extended discussion of the related research, a refined description of the high and low-level attributes and the definition of the relations between them. In addition, the experimental and analysis part was significantly extended by performing experiments with another dataset [19] that contains event logs of different systems in Splunk format, while the previous experiments were performed with network traffic data.

Thus, the contributions of this paper are as follows:

- a taxonomy of the attacker attributes and the specification of the relations between high-level and low-level attributes.
- a methodology for the attacker profile generation that links low-level attributes calculated from raw data and high-level attacker characteristics.
- experiments with a subset of low-level attacker attributes represented by a system event log to understand their applicability to the attacker type definition.

In the future research it is planned to map the selected low-level attributes to the high-level attributes, to extend the list of high-level and low-level attributes and to expand the experiments to enhance the proposed attacker profile. Besides, it is planned to introduce the risk analysis technique using the proposed attacker model and to answer the last question specified above.

The paper is structured as follows. Section 2 classifies the approaches to risk analysis considering the attacker models and concludes with the existing challenges in attacker attribution. Section 3 specifies a formal attacker model, introduces classification of attributes for the attacker model specification, and provides preliminary mapping between low-level and high-level attacker attributes. Section 4 discusses the problem of possible datasets for the attacker’s profiling task, and Section 5 presents the results of the performed experiments. We conclude with a description of future work directions.

## 2. Related Works

In the existing security standards, an attacker is one of the key entities that have to be considered in risk analysis processes. We analyzed a set of studies to understand what approaches exist in the area. Based on the conducted analysis, we outlined four main groups of techniques for attacker specification and modelling in risk analysis tasks [17]:

- attack graph analysis;
- hidden Markov model;
- fuzzy inference;
- attributing cyber attacks using data mining techniques including neural networks, statistics, etc.

*The techniques based on attack graph analysis* represent attacker aims and actions as a set of linked nodes [3,20,21,22,23,24,25,26]. In these techniques, the attacker model (or profile) is usually specified using two characteristics—skills and location. In some cases, motivation, privileges and goals (aims) are also considered. For example, the location can take the values “internal” or “external”, and the skills can take the values “low”, “medium” or “high” [3,23,26]. The set of characteristics included in the attacker profile can be extended and include intent, access, outcome, limits, resource, skill level, objective and visibility [4]. In such models, attacker steps, location and privileges are modelled using attack graph, while other characteristics are usually given on the basis of expert knowledge.

The advantages of such techniques are as follows: (1) they show a list of vulnerabilities that could be exploited by the given attacker; (2) they represent attacker possible paths; (3) they specify the attacker’s possible goals.

The disadvantages of such techniques are as follows: (1) they use expert knowledge to define the probabilities of the next attack action selection, attacker skills and location; (2) in major cases, they use only two attributes to specify an attacker model, namely, skills that could be defined explicitly or implicitly and attacker location; (3) the definition of the probabilities is a complicated process and requires great expertise from the security administrator.

*The techniques based on the hidden Markov model (HMM)* use HMMs for modeling normal behaviour and detect cyber attacks as deviations from this normal behaviour. HMMs are generated on the basis of system states and transitions between them, which are caused by events [5,6,13,27,28]. Each transition is characterized by a probability that is independent from the past, i.e., the behaviour of a process at a given point in time depends only on the state of the process at a previous point in time.

This group of the techniques usually does not use the attacker model explicitly. However, the prediction of the attack goal is carried out on the basis of the most probable transition for the current system state, i.e., the most frequently met sequence of events. The research in [28] differs in that the authors specify the attacker behaviour based on the attacker goals, intention and level of expertise and outline eight profiles of attackers such as criminal groups, insiders, terrorists, hackers, phishers, nations, spyware/malware authors and botnet operators. However, the definition of the HMM presented in their approach does not consider the attacker profile. The authors used attacker profiles to generate different training sets containing 5 types of the malicious behaviour (scanning, enumeration, access attempt, malware attempt, exploitation by denial of service).

The advantages of such techniques are as follows: (1) they allow us to model normal and abnormal behaviour; (2) they allow us to detect insider threats; (3) they link different types of events in one model that is able to reveal trends in attack implementation and is able to detect abnormal attack sequences. The disadvantages of such techniques are as follows: (1) the result strongly depends on the input dataset and the distribution of the events; (2) they do not use the attacker model explicitly.

*The techniques based on fuzzy inference* apply fuzzy logic to produce some averaged description of the parameters used to describe either normal or malicious activities [7,29,30,31]. The fuzzy rules are constructed for classifying the types of malicious activities. These techniques are divided into two broad groups: (1) techniques that use fuzzy inference to detect the type of malicious activity, while the fuzzy rules describe generalized (fuzzy) dependencies between security event attributes; (2) techniques focused on risk assessment that use the attacker profile explicitly as input variables defining the success rate of the attack.

In [32], the authors construct the profiles of the normal user behaviour to detect cyber attacks. They use the following low-level characteristics to specify profiles based on the analysis of the log events: keyboard keys’ sequences, characteristic data sequences retrieved from the pointing device, chosen options, requested network resources, etc. In [33], the authors predict attacker behaviour based on the attack step characteristics. The following parameters characterizing the attack steps and depending on the attacker are used: the required knowledge to perform attack action; the required access to perform attack action (physical or remote); the required user interaction level; and the required skills [33]. The complexity of the attack step (and its attractiveness for the attacker) depends on the values of these four variables. In [14], the authors model the impact of cyber attacks depending on the attacker profile. They describe the attacker profile as a combination of the following three parameters: knowledge, technical resources and motivation. They specify the following six types of attackers: script kiddie; hacker; disgruntled employee; terrorists; industrial spy; and cyber warrior. In [15], the authors try to link attack steps (scanning/reconnaissance, enumeration, exploit by access attempt, exploit by denial of service, exploit by malware attempt) to produce an attacker profile and output the attacker category (criminals; insiders; terrorists; hackers; phishers; nations; spyware/malware authors; bot net operators; amateurs/script kids) depending on the performed steps.

In all aforementioned techniques, the variables describing the attacker profile are linguistic variables that take values from fuzzy sets. The key advantage of the fuzzy logic techniques is an ability to operate with uncertainty, i.e., an ability to describe such fuzzy parameters as motivation or knowledge of the malefactor. The disadvantage of this group lies in the inability to link low-level events to the attributes used to characterize the malefactor profile.

*The techniques based on attributing cyber attacks using data mining techniques* assume the determination of the attack author based on behavioral indicators [8]. Behavioral indicators are represented by a combination of actions and other indicators of malicious activity. These indicators can be atomic or computed. Atomic indicators are discrete pieces of data that cannot be broken down into their components without losing their forensic value. Atomic indicators include IP addresses, email addresses, domain names, and small pieces of text. Computed indicators are similarly discrete pieces of data, but they involve some element of computation. An example is a ‘hash’, a unique signature derived from input data, for instance a password or a program. The hashes of programs running on their network’s computers may match the hashes of programs known to be malicious.

In [34], the authors develop a cyber attacker model profile to predict cyber attacks. The authors define two types of variables including dependent variables (frequency and distribution of attacks, money earned from cyber crime) and independent variables (unemployment rate, level of education, corruption). The authors constructed the attack prediction model linking both types of variables and showed how much variation in the dependable variables they can explain for given values of independent variables. In [9], the authors use honeypot data for risk assessment. The authors define an attacker via a unique tuple (source IP address, operating system, user–agent (protocol), cookies) and consider the attacker score in the risk score. This paper is interesting because the authors made an attempt to link low-level and high-level attacker attributes. Honeypot data are used to calculate skill, resources, motivation, and intention. Further, they integrate skill and resources into the capability rating and integrate motivation and intention into the threat rating. Their combination is used to calculate the total threat score. The authors use the following classes of attackers: guest, external employee, internal employee, activists, state-sponsored, ethical hacker, criminals, cracker and hobby hacker. In [16], the authors propose a method for predicting cyber attack behaviour using recurrent neural networks. They use the dataset obtained from the 2017 Collegiate Penetration Testing Competition to obtain long short-term memory models. The attacker model is considered implicitly here. The used features are as follows: destination port, alert signature, alert category, alert severity, protocols, source port and host.

In [35], the authors analyzed the event log from the 2018 National Collegiate Penetration Testing Competition (CPTC’18) to profile attacker teams. They mapped the team steps represented by the events to the MITRE ATT&CK tactics and techniques.

The last group of studies is the closest to the research direction presented in this paper. However, the challenge of linking raw data with valuable attacker metrics still exists, the feature set is still not specified, the set of metrics that forms the attacker profile is not unified, and the techniques of metrics calculation on the basis of the extracted features should be enhanced. This paper presents the first steps to overcoming these challenges. Namely, we propose a formal attacker model that links raw data and high-level attacker metrics; we classify attacker attributes and make a preliminary attempt to link high-level and low-level attributes. The requirements of the datasets for the experiments are specified, and variants of datasets for attacker attribution are analyzed. The first experiments with a subset of attacker low-level attributes are conducted, and we check if they are applicable for the classification of attacker type.

Table 1 summarizes main advantages and disadvantages of the outlined approaches to attacker specification and modelling and compares suggested approach to the existing ones.

## 3. Attacker Profiling

### 3.1. Research Methodology

In this research, we proceed along our attacker profiling steps. The aim was to specify the attacker model, allowing one to forecast attacker behaviour. The research was conducted as follows:

1. Specify a formal attacker model (or profile) as the set of high-level attributes that are calculated using low-level attributes. The model is given in Section 3.2.
2. Select high-level attacker characteristics as well as the features extracted from network traffic and event logs that can be used for their calculation. The selected attributes are given in Section 3.2.1 and Section 3.2.2.
3. Specify the requirements of the dataset for the experiments and select the dataset. The requirements and the datasets themselves are described in Section 4.
4. Conduct the experiments to check if the features selected in this research, namely, bash commands, allow us to outline different types of attackers. The experiments using different methods are presented in Section 5.

In [18], we conducted the experiments with low-level attributes from the attack traffic gathered during DEFCON 26 CTF [41], namely, the intensity of receiving and sending network packets; bytes per time interval or the intensity of receiving and sending bytes; TCP dialogs; TCP-points from network traffic, i.e., pairs of IP addresses and port; IP-points; number of ports; number of protocols; IP dialogs; and IP-address. It was concluded that while these characteristics allow us to differentiate between the attackers’ skill levels, they are not sufficient for the attacker profile specification.

### 3.2. Attacker Model and Classification of Attributes

This research is focused on attacker model specification and the analysis of its application and usefulness for risk analysis tasks. We aim to link features obtained from the raw data (i.e., logs and network traffic) to the attacker characteristics (or attributes). Thus, the attacker model At is specified as follows [18]:

(1) At={HF,LF,Relations},

where $HF={\left\{hf_{i}\right\}}_{i=0}^{n}$—high-level attacker characteristics, *n*—number of high-level attacker characteristics; $LF={\left\{lf_{j}\right\}}_{j=0}^{k}$—low-level attacker characteristics derived from the raw security data, and *k*—number of low-level attributes. A mapping Relations:LF⟶HF maps low level attributes to high-level attributes.

The attributes of each class are divided into semantically meaningful groups describing different aspects of attacker behaviour. These groups are described in the next subsection.

#### 3.2.1. The High-Level Attributes

The high-level attributes are quite abstract notions that cannot be derived directly from raw data while monitoring the system under the analysis. These attributes are usually evaluated using expert methods and are therefore often subjective.

**Figure 1:**
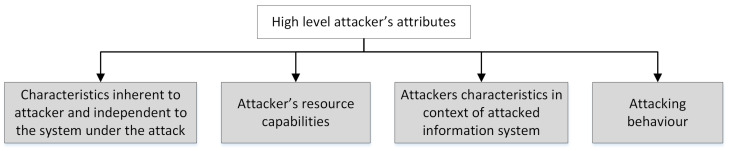
Main groups of the high-level attributes.

The groups for the high-level attributes are shown in Figure 1 and described below in detail.

The **first group** incorporates inherent attacker characteristics:

- Skills (or level of expertise)—this characteristic represents the attacker’s ability to implement complex attacks and use complex tools, experience and knowledge in the area, and ability to cover up the traces and stay in the system undetected for a long time (skills can be scored using different scales, for example as high, medium or low). In the scope of risk analysis tasks, higher skills indicate that the attacker can implement more complex attacks and bypass more complex security measures for a shorter time interval.
- Motivation—this characteristic represents the attacker’s desire to implement an attack successfully and can be represented by the number of attack attempts, time spent on the attack, and resources spent on the attack (motivation can be scored using different scales, for example as high, medium or low). In scope of risks analysis tasks, higher motivation indicates that the attacker will not stop in spite of security measures.
- Intention—this characteristic represents the attacker’s expectations from the successful attack implementation (for example, financial gain). In the scope of risk analysis tasks, this characteristic can indicate what attack path the attacker will choose.

The **second group** characterizes the attacker’s capabilities:

- Used resources—this characteristic represents resources available to the attacker to implement the attack (for example, expensive equipment). Used resources and skills are connected in terms of the complexity of used resources. In terms of risks analysis, resources indicate whether the attacker can implement more complex attacks and bypass more complex security measures for a shorter time interval.

The **third group** connects the attacker and the system under attack:

- Location—this characteristic represents the attacker position relative to the system (for example, outside the system, inside the system, and, if inside, where exactly the attacker is). In the scope of risks analysis, the task location indicates whether the attacker is close to the critical assets and what paths the attacker can select. It is connected to the system via the objects the attacker has access to, type of access and privileges and detected activity (events and incidents).
- Privileges—this characteristic represents the attacker’s privileges in the system (for example, user or administrator). In the scope of risks analysis, task location indicates whether the attacker is close to critical assets and what paths the attacker can select. It is connected to the system via the objects the attacker has access to, type of access and privileges, and detected activity (events and incidents).
- Goals (aims)—this characteristic represents the attacker’s goal. It differs from the “intention” characteristic by the fact that the goal is specified in terms of the system under attack (for example, elevate privileges on the server). In the scope of risks analysis, the task indicates what paths the attacker will select. It is connected to the system via the objects the attacker aims to compromise and the type of privileges the attacker aims to obtain.
- Access—this characteristic represents the type of the attacker’s access to the system’s objects (for example, physical or remote). In the scope of risks analysis, this indicates what paths the attacker can select. It is connected to the system via the objects the attacker has access to, type of access, and detected activity (events and incidents).
- Knowledge—this characteristic represents the attacker’s knowledge of the system under attack (for example, system topology). In the scope of risk analysis, this indicates what paths the attacker selected before and what actions the attacker has already implemented that, in turn, allows us to estimate the attacker’s skills. It is connected to the system via the objects the attacker has accessed before, type of access and privileges, and detected activity (events and incidents).

The **fourth group** connects the attacker and the attack:

- Attack steps—this characteristic represents the type of the attacker’s actions in the system (for example, reconnaissance or exploit). In the scope of risk analysis, this indicates what paths the attacker can select. The attacker’s steps are also connected to the system under attack, namely, with the “location”, “access” and “privileges” characteristics and the detected activity (network traffic, events and incidents).

#### 3.2.2. The Low-Level Attributes

The low-level attributes can be calculated directly from the raw data gathered while monitoring the system under analysis. Thus, they do not depend on expert assessments and can be considered objective.

The low-level attributes can be classified by their source. Currently, we outline the following sources: event logs and network traffic. Thus, low-level attributes are represented by characteristics of events or network traffic.

**Figure 2:**
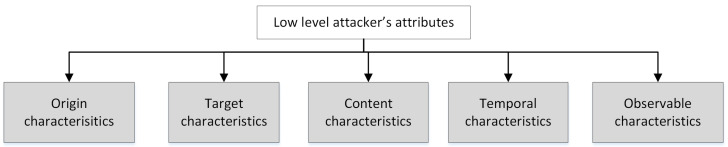
Main classes of the low-level attributes.

In [9] Fraunholz et al. proposed the following classification of network traffic characteristics: origin characteristics, target characteristics, content characteristics and temporal characteristics. We extended this classification by including an additional class—observable attack characteristics. This classification could be applied both to the network- and log-based characteristics. Their taxonomy is presented in Figure 2, and their structure is discussed below in detail.

**Origin characteristics** describe the attack (or normal action) source used by an attacker. They include: ports obtained from network traffic or events log; IP addresses from network traffic or events log; IP-points from network traffic; TCP-points from network traffic (pair IP address and port); user–agent (protocol) from network traffic or events log; URL obtained from network traffic [9]; e-mail addresses from network traffic/log [9]; domain names from network traffic/log; operating system from network traffic/log; UserID from network traffic/log; {cookies} from network traffic [9].

**Target characteristics** describe the attacker goal or the destination of the attack (or normal action). They include: IP addresses from network traffic/log; domain names from network traffic/log; operating system from network traffic/log; vulnerability (can be defined based on other features of network traffic or events log, namely, operation system or protocol); host from network traffic/events log; port obtained from network traffic or events log.

**Content characteristics** specify the attack (or normal action) content (or payload). They include small pieces of text from network traffic/log; hash from network traffic/log; keyboard keys’ sequences from event logs [32]; commands from network traffic [9]; files from network traffic [9]; exploits from network traffic [9]; chosen options from event logs [32]; requested network resources from event logs [32].

**Temporal characteristics** characterize frequency and time aspects of attacks (or normal actions) in the selected time interval. They include frequency of attacks based on network traffic or log [34]; distribution of attacks based on network traffic or log [34]; frequency of alerts based on events log; distribution of alerts based on events log; inter-arrival time [9]; session duration [9]; files per time interval [9]; packets per time interval [9] or the intensity of receiving and sending the packets; bytes per time interval or the intensity of receiving and sending bytes; TCP dialogs between TCP-points; IP dialogs between IP-points; commands per time interval [9]; inter-session time [9]; sessions per time interval [9]; number of ports; number of protocols; number of used vulnerabilities; number of used exploits.

**Observable attack characteristics** incorporate observable characteristics of the attack not included in the four aforementioned classes. They include: alert signature from events log [16]; alert category from events log [16]; alert severity from events log [16]; sequence of attack actions; average alert severity.

Obviously, the values of the low-level characteristics highly depend on the available source raw data. They require the establishment of data pre-processing procedures specific to different data sources.

**Figure 3:**
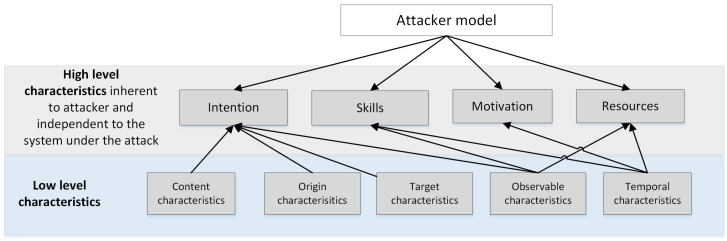
Overview of the links between high-level attributes and the related sets of low-level attributes.

The next step is to establish dependencies between the low-level attributes and high-level attributes. This is a crucial and complicated step, as it is necessary to consider existing relations between low-level attributes and high-level attributes. At the moment, we defined the set of attributes that may be used to calculate the high-level attributes as belonging to the first group of attributes, i.e., attributes inherent to the attacker. Table 2 shows this mapping in detail, while Figure 3 gives an overview of the defined links between the groups of low-level attributes and specific high-level attributes.

The established dependencies between attributes are quite complex, and many low-level attributes are used to define different high-level attributes simultaneously; however, their impact on the values may vary depending on the origin of the high-level attribute. Defining algorithms for calculating the high-level metrics (or attributes) based on them is a rather challenging task. Considering the fact that the existing relations between the attributes could be nontrivial and nonlinear, a possible solution lies in the application of machine learning techniques. The subsequent sections investigate the problem of the applicability of machine learning techniques to define links between low-level and high-level attributes and discusses possible datasets that could be used to train an analysis model.

## 4. Data Sources for Attacker Profiling

In general, the object attribution is a classification task and requires labeled data that contain data samples with labels specifying the type (or class) of a given sample. Such data are used to train analysis models that can further determine the class of a new sample with some level of confidence (or probability). The selection of features for model training is implemented on the basis of a thorough analysis of the characteristics of the training set, including the possible correlation of features with class values.

Thus, in order to implement attacker attribution and the fine-tuning of features used in the analysis process, the following are required:

1. the training dataset must contain a lot of attack actions against one information system performed by the attackers with different skills, resources, intentions and motivations;
2. the dataset has to be labeled, as we need to know what actions were performed by which attacker.

Although there is no such pre-prepared dataset to which to attribute a malefactor attacking style, we think that the possible solution to this problem is the usage of datasets collected during capture the flag (CTF) competitions, as in general they satisfy the requirements listed above. The modeled infrastructure is common for all participants, and that is why the network traffic and events generated by each team depends on skills, knowledge and computation resources and can be used to characterize individual or team attacking styles. The only problem is that such datasets do not contain explicit labels for high-level attacker features. However, some information about contest winners can be used to identify the most efficient teams or at least the number of efficient teams and their scores. The scores obtained by teams could be used to characterize a list the their skills as well as their resources.

The following datasets were outlined for our experiments:

- network traffic from DEFCON 26 CTF [41];
- dataset from the National Collegiate Penetration Testing Competition 2019 (National CPTC 2019) that contains event logs from different systems [19].

The DEFCON 26 CTF dataset and National CPTC 2019 were generated during CTF competitions and differ in that the DEFCON 26 CTF dataset is represented by PCAP packets while the National CPTC 2019 data contain event logs, which means that it is possible to evaluate features of different types and origin.

During the DEFCON 26 CTF, 24 teams/participants competed at the final stage to exploit vulnerabilities in the information system deployed for the CTF, to compromise opponents’ computers and protect their own assets. The dataset contains network traffic collected over nearly 2 days of competition [19].

The National CPTC 2019 dataset was gathered during the attacks against the developed fictitious organisation (DinoBank) imitating a financial institution. The dataset contains the event logs of different systems of DinoBank in Splunk format [42].

In [18], we conducted the first experiments using the network traffic from DEFCON 26 CTF [41]. We selected such attributes as the intensity of receiving and sending network packets, bytes per time interval, number of different TCP dialogs between TCP-points, number of TCP-points from network traffic, IP-points etc. The results of the experiments showed that these attributes allowed us to differentiate between different behavioural patterns, but they are enough to determine the skill level of the team. Some teams who received high scores were clearly seen as outliers, but the winner did not exhibit any extraordinary network behavior and was always among the teams with average scores.

The section below presents the results of the experiments performed with the National CPTC 2019 dataset and discusses its applicability to the attacker attribution task.

## 5. Experiments with the Selected Dataset for Feature Selection

In this paper, we present the next series of experiments using the dataset from the National Collegiate Penetration Testing Competition 2019 (National CPTC 2019) [19] and focus on the analysis of the bash commands as a possible source for attacker attribution. Thus, the following hypothesis was made: the bash-history can profile the attacker, and thus we selected the bash-history as low-level attributes.

The experiment incorporated the following stages:

1. dataset collection.
2. dataset preprocessing.
3. dataset analysis.
4. model training and experiments.

**The data collection, analysis and preprocessing.** The National Collegiate Penetration Testing Competition 2019 (National CPTC 2019) [19] was a 2-day event in which national and international teams had to hack a virtual bank called Dinobank. In 2019, 66 teams from 6 regions participated in CPTC-19. Each region was represented by up to 12 teams, the only exception was the foreign region, which was represented by 4 teams only.

The event log includes all events collected from all machines across all teams. Thus, the initial dataset contains events from 18 different OS Linux services such as ftp server daemon, iostat, df, network stat, etc., and 6 OS Windows utilities. In order to use the CPTC-19 dataset for data analysis, the initial dataset was transformed from Splunk format to CSV format, and the records were grouped by team and source type. In this research, we focused on the analysis of the bash commands represented by nominal attributes such as team_name and bash-history commands.

However, it is impossible to identify an effective attack or to say that a particular team won the competition due to the lack of the information on the teams’ scores. It is possible to extract information only about one team; however, there is no information regarding their position and scores in the competition from the data provided. Therefore, we re-formulated the problem of the attacker profiling to the task of team attribution; thus, we analyzed whether it possible to differentiate between different team attacking behaviour by analyzing bash commands.

The number of commands entered by five teams from one region in the competition are shown in Table 3.

**Figure 4:**
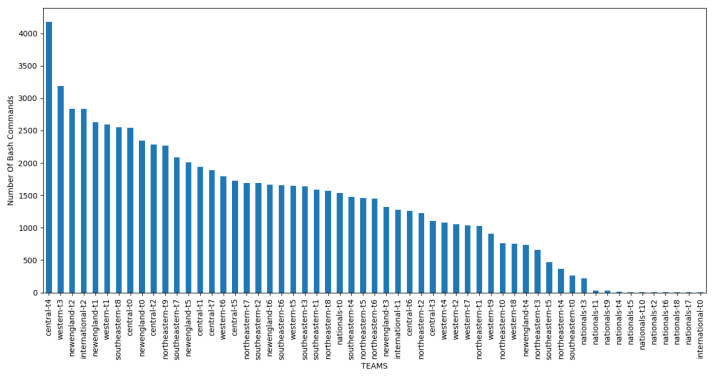
Distribution of number of commands entered per team.

To prepare the data for training and testing, missing values from the collected CPTC 2019 data were first filled with 0s and bash-history logs were grouped by team name. In the second step, all team member names from the original long-name labels and host names were refactored to form one label associated with one team. For example: *nationals-t0-corp-coins-01*, *nationals-t0-corp-web-03* and *nationals-t0-bank-heads-01* were all renamed to *nationals-t0*. Table 4 shows the results of the name unification procedure. Figure 4 shows the number of commands entered by each team. It can clearly be seen that team ’*central-t4*’ entered the largest number of commands in the event, followed by teams ’*western-t3*’ and ’*newengland-t2*’.

In the preprocessing stage, we also removed the timestamps of the bash commands.

Further data pre-processing included label encoding and text vectorizing. To obtain binary representation for each team, one-hot encoding was applied; for example, team *nationals-t0* was assigned vector 11 and team *international-t1* was assigned vector 9. Table 5 and Table 6 show the key steps of these transformations for the selected teams.

To process the raw ’bash-history’ commands, we applied a word tokenization technique using two modules that are described below:

- *Fit_on_texts*: Based on the frequency of bash_history texts, a dictionary with indexes was created using Keras Fit_on_texts. Each word was assigned an integer value based on their repetition frequency, with highly repeated words having the lowest integer value (i.e., ls command is 0). The resulting output can be seen in Table 7.
- *Text_to_sequences*: Each word from the input (bash_history) logs was replaced with the index from the dictionary made from *fit_on_texts*, as shown in Table 8.

**Figure 5:**
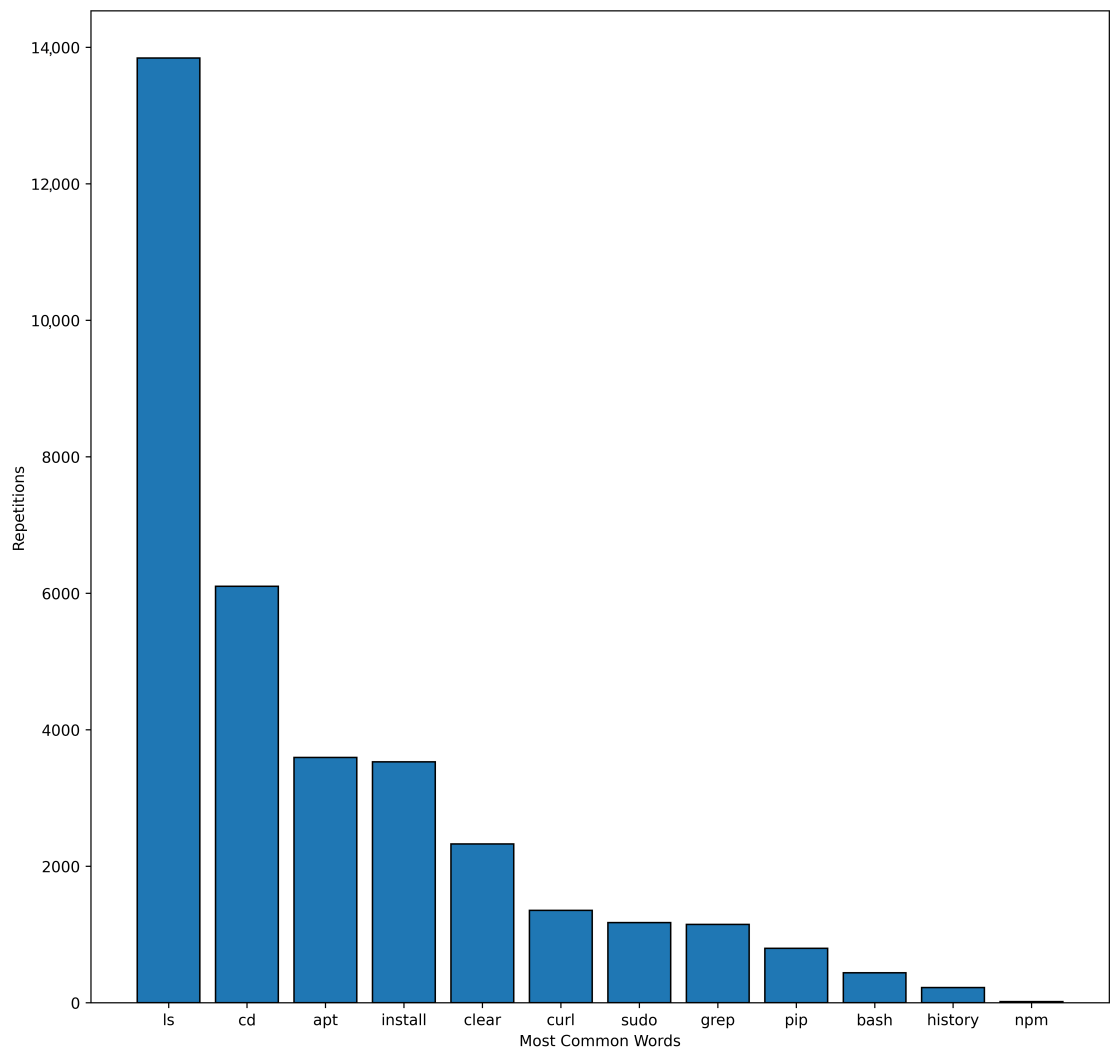
The most frequently met word tokens (bash commands).

Figure 5 shows the most frequently met tokens that represent bash commands and their parameters.

**Model training and experiments.** A long short-term memory (LSTM) neural network model was designed to take into account lags of unknown duration for each bash-history command input and the time-series format of the data. An additional embedding layer was added to the sequential LSTM model to learn the index sequences in Table 8 as embeddings. The configuration of the model is provided in Table 9. LSTMs are capable of learning long-term dependencies and are designed to avoid this problem. LSTMs consists of chain-like structures just like RNNs (recurrent neural networks) except for the repeating module, which, instead of having a single layer, consists of specially interacting layers. There are three different gates in an LSTM cell, which are represented as follows:

**Forget Gate:** This takes the inputs $h_{t-1}$, $x_{t}$ and applies a sigmoid function σ to give an output distribution between 0 and 1 for each number in the cell state $C_{t-1}$

$$f_{t}=\sigma \left(W_{f}\cdot \left[h_{t-1},x_{t}\right]+b_{f}\right).$$

**Input Gate:** In this layer, two operations are performed, first a sigmoid layer outputs a value to be updated and second a tanh layer creates a vector ${\tilde{C}}_{i}$ to be added to the cell state $C_{t-1}$

$$\begin{array}{cc} i_{t} & =\sigma \left(W_{i}\cdot \left[h_{t-1},x_{t}\right]+b_{i}\right)\\ {\tilde{C}}_{t} & =\tanh\left(W_{C}\cdot \left[h_{t-1},x_{t}\right]+b_{C}\right). \end{array}$$

**Output Gate:** Finally, in the output gate, the old cell state $C_{t-1}$ is updated to the new cell state $C_{t}$ by multiplying the old state by $f_{t}$ and adding $i_{t}*{\tilde{C}}_{t}$.

$$C_{t}=f_{t}*C_{t-1}+i_{t}*{\tilde{C}}_{t}.$$

**Figure 6:**
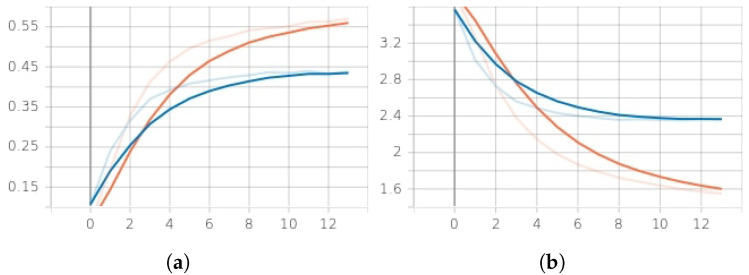
Training/validation accuracy vs. loss per epoch (12 epochs). (**a**) Training (red)/validation (blue) accuracy. (**b**) Training (red)/validation (blue) loss.

Figure 6 demonstrates the results of the learning. The model converges to over 50% accuracy in 10 epochs. The overall accuracy of the LSTM classifier achieves 61% for the dataset compared to other algorithms such as SVM (support vector machine) and random forest classifier, as shown in Table 10. The training loss fails to go below 1.2. The learning curve for the model is represented in Figure 6a,b.

**Discussion.** Let us analyze the training results first. The parameters of the model training are given in Figure 6a,b. The bash-history logs were collected from different attacker teams with very similar attack patterns using mostly similar commands. The word index dictionary (see Table 7) has 11,238 unique words. The longest sentence in the index sequence has a length of 647. As the result, a single sample size is 647×1, the total number of samples is 42,543 and the total number of team labels is 66. The neural network takes these data as an input. Large dimensions of data samples and a large number of teams make it difficult for the model to learn useful embedding and give high classification accuracy, i.e., above 61%. Additionally, the similarity of the sequences in the data under analysis complicates the identification and discarding of the less useful sequences. It should be noted that at least 10 out of 66 teams in the competition used the same command twice, and their behavior is not distinguishable from each other. To enhance the classification accuracy, the teams with similar activities can be removed from the data during the pre-processing stage to reduce the number of labels.

We compared the obtained results with other classification algorithms. Our comparison is presented in Table 10. Considering the results of the conducted experiments with low-level attributes and event logs, it can be concluded that the teams exhibit very similar behavior.

There are the following research gaps that will be eliminated in future research to enhance the classification accuracy and attacker profile: (1) to enhance the accuracy, the data could be divided by region [43] before applying a classification algorithm; (2) the timestamps are excluded from the analysis, their consideration will allow us to analyze the consequences and frequency of commands; (3) to map high-level attributes such as “attacker skills” to the low-level attributes, we obviously need additional data, i.e., we need to outline additional low-level attributes.

It should be noted that compared to other studies, the suggested approach differs in terms of the method of specifying the attacker model. While analysing the related research, the authors found a lack of formalization and systematization of the attacker models. The authors’ goal is the clear detection of the low-level attributes and mapping them to the high-level attributes characterizing the attacker for further applications in risk analysis tasks. In Table 1, we provide the main differences between our approach and existing approaches. The approach based on the attack graph analysis mainly uses high-level metrics not mapped to the raw data and is thus rather subjective. The HMM-based approaches apply high-level metrics that are not mapped to the raw data, and their set is not unified. The approaches based on fuzzy inference can use both high-level and low-level metrics, but they are limited to the detection of abnormal users’ behavior and are highly dependent on the correct synthesis of information flows. The attack attributing approaches are the closest to our approach. They attempt to link the raw data and high-level metrics, but techniques for the calculation of specific metrics require further development, and specific classes of attackers are not considered. The approach provided in this paper considers step-by-step selection and the linking of the high-level and low-level metrics to outline different attacker profiles. Therefore, it can be said that the training data bash_history gives good enough results at 61% classification accuracy with 66 team labels and is appropriate to train machine learning models such as LSTM for classifying different team labels from the competition.

## 6. Conclusions

This paper investigated the attacker profile concept. Several challenges related to attacker profile specification and its applications in risk analysis are outlined. An attacker model specification method incorporating high-level and low-level attributes and the connections between them is proposed. The classes of attributes and the preliminary mapping between high-level and low-level attributes were outlined. Based on the outlined requirements of the datasets for the experiments, two datasets suitable for attacker profiling were selected. This paper focused on a dataset that incorporated event logs.

It should be noted that due to the selected dataset features, it is possible to extract information only about a team; however, there is no information relating to their position and scores in the competition. Therefore, it is possible to differentiate between different teams’ attacking behaviour by analyzing their bash commands. To derive an attacker profile, additional parameters should be analyzed, and specific datasets are required. Therefore, we re-formulated the problem of attacker profiling to the task of the team attribution; thus, we analyzed whether it possible to differentiate between different team attacking behaviors by analyzing bash commands.

Different analysis techniques were implemented to preprocess the dataset. The LSTM model was trained to classify the attacker profiles. The provided experiments demonstrated that bash history logs allow us to differentiate between the selected attacker teams. While the obtained accuracy is 61%, this represents a good classification accuracy for 66 teams. The bash history logs collected from different attacker teams are very similar to each other. It is likely that the accuracy of classification will increase if the teams with all or mostly similar activities are removed and the number of labels is reduced to 2–5 teams.

It is possible to conclude that the nominal datasets such as “bash_history” logs, collected across different attackers from CPTC 2019, give significant results for classification across 66 teams. The accuracy of the model can be improved if only 2–3 teams are considered. The obtained results are the basis for the accurate specification of the attacker profile in terms of interconnected high-level and low-level attributes.

In future work, we plan to extend the set of low-level attributes and enhance the mapping between the low-level and high-level attacker attributes. Future steps will include the development of the algorithms for calculating high-level attacker metrics on the basis of low-level attributes, research into the last question posed (do we really need the attacker model for risk analysis?) and the development of techniques for the application of the attacker model in risk analysis techniques if the answer to this questions is yes.

## Figures and Tables

**Table 1:** Comparison of the existing approaches with the proposed approach.

Approach	Input Data Source	Type of Metrics	Advantages & Limitations
Attack graph analysis [3,20,22,23,24,36,37,38]	Network topology, software and hardware configuration, relationships between users and services, vulnerabilities	High-level (attacker skills, location)	Focus on the vulnerabilities existing in the system. Extensive usage of the expert knowledge to quantify metrics.
HMM-based approach [5,13,14,15,27,28,39,40]	Events generated by honeypots and network traffic with emulated attacks	High-level (goals, intention, level of expertise)	There is no link of the low level events to high-level attributes. Not unified (in terms of attacker profile and metrics).
Fuzzy inference [32,33]	Can be high-level abstract data or qualitative attributes of the log events	High-level (skills, knowledge, access (location), interaction) or low-level (keyboard keys’ sequences, characteristic data sequences)	Deals with uncertainty in the data. Limited with detection of abnormal user’s behaviour. Highly depends on the correct synthesis of information flows
Attack attributing [8,9,16]	Network traffic data	High-level (skill, resources, motivation, intention) and low-level (IP addresses, email addresses, domain names, small pieces of text, hash, cookies etc.)	Attempt to link raw data and high-level metrics. Techniques for calculation of specific metrics require further development. Specific classes of attackers are not considered.
**This approach**	**Network traffic data and event logs**	**High-level (skills, education etc.) and low-level (the intensity of receiving and sending network packets; bytes per time interval or the intensity of receiving and sending bytes; TCP dialogs; TCP-points from network traffic, i.e., pairs IP address and port; IP-points; number of ports; number of protocols; IP dialogs; IP-address; bash commands etc.)**	**Linking raw data (low-level metrics) and high-level metrics to profile the attacker. In progress.**

**Table 2:** Mapping of the low-level attacker attributes to high-level attributes.

High-Level Attributes	Group of Low-Level Attributes	Low-Level Attributes
Skills	Observable attack characteristics that characterize ability to cover up the traces. It is assumed that in case of higher skills the incidents rate will be lower and location in network will be deeper.	Frequency of alerts (malware detection rate), Distribution of alerts
	Temporal characteristics that could be used to characterize tools complexity (also used scripts and commands should be considered)	Number of used exploits (known exploits, exploits with high complexity)
	Temporal characteristics that characterize attacker experience and knowledge (here focus is done on the complexity of actions, their severity and performance)	Frequency of alerts, Distribution of alerts, Frequency of attacks, Distribution of attacks, Command per time interval, Packets per time interval, Bytes per time interval, Inter-arrival time, Session duration, IP dialogs, TCP dialogs, Files per time interval, Inter-session time, Sessions per time interval, Number of ports, Number of protocols, Average alerts severity, Number of used vulnerabilities, Number of used exploits.
Motivation	Temporal characteristics	Frequency of alerts, Frequency of attacks, Command per time interval, Packets per time interval, Bytes per time interval, Inter-arrival time, IP dialogs, TCP dialogs, Files per time interval, Inter-session time, Sessions per time interval, Number of ports, Number of protocols, Number of used exploits.
Intention	Origin and Target characteristics	IP addresses from network traffic/log. Domain names from network traffic/log. Operating System from network traffic/log. Host from network traffic/events log. Port obtained from network traffic or events log, Requested network resources
	Observable attack characteristics	Alert signature, Alert category, Vulnerability, Exploits
	Content characteristics that describe system state after attack action, resources state after attack action (e.g., modified, removed)	Alert signature, Alert category, Alert severity, Vulnerability, Small pieces of text, Hash, Commands, Exploits
Resources	Attack coverage	Distribution of attacks, Number of ports, Number of protocols, Number of used exploits
	Temporal characteristics	Frequency of attacks
		Inter-arrival time, File per time interval, Packet per time interval, Bytes per time interval, Command per time interval, Inter-session time, Sessions per time interval

**Table 3:** Statistics on raw categorical data—Bash History from the CPTC 2019.

Team Name	Num. of Unique Commands
central_team0	1480
central_team1	1368
central_team2	1308
central_team3	483
central_team4	1715
central_team5	1098

**Table 4:** Preprocessed team label.

Before		After	
**_raw**	**host**	**_raw**	**host**
exit	world-build-t0-vdi-ns01	exit	world-build-to
vim db.dinobank.us	world-build-t0-vdi-ns01	vim db.dinobank.us	world-build-t0
Is	world-build-t0-vdi-ns01	Is	world-build-t0
cd/var/cache/bind/	world-build-t0-vdi-ns01	/var/cache/bind/	world-build-t0
nc -lvnp 40,000	western-t9-vdi-kali05	nc -lvnp 4444	western-t9
ifconfig	western-t9-vdi-kali05	nc -nlvp 100	western-t9
ssh 10.0.1.33	western-t9-vdi-kali05	msfconsole	western-t9

**Table 5:** One-hot representation of team names.

Team Label	Central-t0	International-t0	Nationals-t0	New-England-t0	North-Eastern-t0	South-Eastern-t0	Western-t1
0	1	0	0	0	0	0	0
8	0	1	0	0	0	0	0
11	0	0	1	0	0	0	0
22	0	0	0	1	0	0	0
29	0	0	0	0	1	0	0
39	0	0	0	0	0	1	0
48	0	0	0	0	0	0	1

**Table 6:** Team names with corresponding labels.

Team Name	Label
central-t0	0
international-t0	8
nationals-t0	11
newengland-t0	22
northeastern-t0	29
southeastern-t0	39
western-t1	48

**Table 7:** Index representation of words in bash-history.

Word Index
’install’: 12
’cat’: 13
’cd’: 14
’ssh’: 15
’sudo’: 48
’cmd’: 987
’psexec’: 966

**Table 8:** Index sequence representation of data.

Bash-History Data	Index Sequences
[’grep -ri 8089 *’]	[40, 1962, 284]
[’clear’]	[526]
[’cd etherex/frontend/’]	[14, 1963, 3653]
[’ls’]	[60]
[’rm -rf tmp/’]	[93, 487, 206]

**Table 9:** LSTM configuration.

Parameter	Value
Type	Sequential
Number of LSTM neurons	64
Dropout	0.7
Loss	CCE (Categorical Cross-Entropy)
Optimizer	Adam
Batch-Size	100
Epoch	20
Activation	Softmax
Test/Train Split 25%	
Additional Layer	Embedding Layer

**Table 10:** Comparison with other machine learning algorithms.

Algorithm	Training Accuracy (%)	Validation Accuracy (%)
SVM-Classifier	25	14
Random Forest	23.2	15
Ours—LSTM Classifier	**61**	**48**

## Data Availability

https://github.com/ashishjv1/CyberAttack_Profiling (accessed on 27 January 2023).

## Abbreviations

The following abbreviations are used in this manuscript:

LSTM	Long short-term memory
HMM	Hidden Markov model
CTF	Capture the flag
RNN	Recurrent neural networks
CCE	Categorical cross-entropy
SVM	Support vector machine
